# Editorial: Robotic Manipulation and Capture in Space

**DOI:** 10.3389/frobt.2022.849288

**Published:** 2022-02-02

**Authors:** Evangelos Papadopoulos, Farhad Aghili, Ou Ma, Roberto Lampariello

**Affiliations:** ^1^ Control Systems Lab, School of Mechanical Engineering, National Technical University of Athens, Athens, Greece; ^2^ Space Exploration, Canadian Space Agency (CSA), Montreal, QC, Canada; ^3^ Intelligent Robotics and Autonomous Systems Lab, College of Engineering and Applied Science, University of Cincinnati, Cincinnati, OH, United States; ^4^ Institute of Robotics and Mechatronics, German Aerospace Center (DLR), Cologne, Germany

**Keywords:** space robotics, on-orbit servicing, robotic capture on orbit, manipulation in orbit, ground testing of space robots

Τasks such as inspecting, refueling, upgrading, repairing, or rescuing satellites, removing of orbital debris, and construction and maintenance of large orbital assets and infrastructures are important for maintenance of space infrastructure on orbit. Until now, all notable servicing tasks have been performed at Low Earth Orbit (LEO) by astronaut Extravehicular Activities (EVAs). However, these are risky, costly, slow, and sometimes unfeasible operations. EVAs can be replaced by robotic on-orbit servicing (OOS), during which the tasks are performed by *space manipulator systems* (SMSs), also called *chasers* or *servicers* in the literature. These consist of a satellite base equipped with one or more *robotic manipulators* (arms) endowed with grappling devices on them, and driven by a vision system, which enables the capture of a *target (client)* satellite. An SMS also can be a large servicing manipulator mounted on a space facility.

This Research Topic is focused on manipulation and capture on-orbit, and on aspects related to these activities. Therefore, it includes work related to the dynamics of rigid and flexible SMS, the associated contact dynamics, the identification methods for space systems, the pose and state sensing needed for monitoring and control, the motion planning methods for grasping a target, the feedback control methods during motion or interaction tasks, and the ground testing testbeds for such systems.

The Research Topic includes five articles. In *Estimation of Vibration Characteristics of a Space Manipulator from Air Bearing Supported Test Data*, Li et al., study theoretically and experimentally an issue related to planar experimental testing testbeds, which use air bearings to support vertically a scaled SMS and create a zero gravity environment on the plane. The authors point out that air bearings influence the dynamics behavior of a scaled SMS, and therefore its apparent joints’ stiffness and damping, its natural frequencies, and its vibration response. The authors present a set of procedures to remove the air bearings influence and identify the true equivalent joint stiffness and damping from the test data of a motor-braked system. The inertial properties are identified, and the equivalent joint stiffness and damping are determined using a genetic algorithm. The true vibration characteristics of the manipulator are estimated by removing the additional inertia caused by the air bearings.

In *On-Orbit Robotic Grasping of a Spent Rocket Stage: Grasp Stability Analysis and Experimental Results*, Mavrakis et al., studied the grasping of a spent rocket stage, analyzed the grasp stability, and presented experimental results. A novel methodology for evaluating the stability of a spent rocket stage robotic grasp is presented based on the calculation of an Intrinsic Stiffness Matrix of a 2-fingered grasp of an Apogee Kick Motor nozzle and a stability metric is defined as a function of the local contact curvature, material properties, applied force, and target mass. The stability metric is evaluated in a V-REP simulator and in two real robot experiments, using several grasps and pulling profiles. Also, a sensitivity analysis is performed to demonstrate how a variation on the grasping parameters affects grasp stability.

In *Autonomous Robots for Space: Trajectory Learning and Adaptation Using Imitation*, Ashith Shyam et al., employ imitation in trajectory learning and adaptation of manipulators on a free-floating spacecraft, to provide more autonomy to SMS. A redundant 7-DoF robotic arm is mounted on a small spacecraft dedicated for debris removal, on-orbit servicing and assembly, autonomous and rendezvous docking. The motion of a redundant 7-DoF robotic arm induces reaction forces on the spacecraft and as a result, its Attitude Determination and Control System (ADCS) takes corrective actions. The method developed finds the trajectory that minimizes the attitude changes and therefore reduces the power and fuel consumption. To determine the trajectory, a cost term is defined and the trajectory minimizing this cost is considered the optimal one. The method employs trajectory learning offline; the learned trajectory distribution can be used for planning in unseen situations by conditioning the probabilistic distribution.

In *A Robust Observation, Planning, and Control Pipeline for Autonomous Rendezvous with Tumbling Targets*, Albee et al., focus on the challenging issue of autonomous rendezvous with tumbling targets. They propose a complete pipeline of rendezvous with such targets, starting from a standoff estimation point to a mating point fixed in a rotating target’s body frame. A novel visual estimation algorithm is applied for remote standoff target state estimation, and a motion planning algorithm is employed producing a look-up table parsed on-orbit using the estimation data. An uncertainty characterization method is demonstrated which propagates the uncertainty in the target’s tumble uncertainty to provide disturbance bounds on the motion plan’s reference trajectory. This uncertainty bound is provided to a robust tube model predictive controller, providing tube-based robustness guarantees on the system’s ability to follow the reference trajectory translationally. The authors present the combination and interfaces of the developed methods, and discuss some of the practical implications of their use on NASA’s Astrobee free-flyer. Simulation results are included.

The last article is a comprehensive survey by Papadopoulos et al., entitled *Robotic Manipulation and Capture in Space: A Survey*. The survey addresses fundamental aspects of manipulation and capture, such as the dynamics of SMS, the contact dynamics between manipulator grippers/payloads and targets, and the methods for identifying properties of SMSs and their targets. Also, it presents recent work of sensing pose and system states, of motion planning for capturing a target, and of feedback control methods for SMS during motion or interaction tasks. The article also reviews major ground testing testbeds for capture operations, and several notable missions and technologies developed for capture of targets on-orbit.

The articles included in this Research Topic provide good exposure to issues related with manipulation and capture of targets on-orbit, and with SMS ground testing. It is expected that these studies will contribute towards the sustainable use of space and the proliferation of robotic systems on orbit, capable for servicing of satellites and large orbital assets, and for capture of unknown space debris.

